# Species Diversity of Edible Mushrooms I—Four New *Laccaria* Species from Yunnan Province, China

**DOI:** 10.3390/jof11030189

**Published:** 2025-02-28

**Authors:** Song-Ming Tang, Guo Zhao, Kai-Yang Niu, Rui-Yu Li, Feng-Ming Yu, Samantha C. Karunarathna, Lin Li, Kevin D. Hyde, Xi-Jun Su, Zong-Long Luo

**Affiliations:** 1 College of Agriculture and Biological Science, Dali University, Dali 671003, China; tang202205@gmail.com (S.-M.T.); 15117569645@163.com (G.Z.); niukaiyang@126.com (K.-Y.N.); lry1156960778@163.com (R.-Y.L.); linlin19870311@163.com (L.L.); luozonglongfungi@163.com (Z.-L.L.); 2 Key Laboratory for Plant Diversity, Biogeography of East Asia, Fungal Diversity and Green Development, Kunming Institute of Botany, Chinese Academy of Sciences, Kunming 650201, China; fm_yu2018@163.com; 3 Center for Yunnan Plateau Biological Resources Protection and Utilization, College of Biologyand Food Engineering, Qujing Normal University, Qujing 655011, China; samanthakarunarathna@gmail.com; 4 Center of Excellence in Fungal Research, Mae Fah Luang University, Chiangrai 57100, Thailand; kdhyde3@gmail.com; 5 Department of Botany and Microbiology, College of Science, King Saud University, Riyadh 11495, Saudi Arabia; 6 Co-Innovation Center for Cangshan Mountain and Erhai Lake Integrated Protection and Green Development of Yunnan Province, Dali University, Dali 671003, China

**Keywords:** four new species, *Hydnangiaceae*, multi-gene, novel species, phylogenetic analysis, taxonomy

## Abstract

As symbiotic mycorrhizal associates, species within the genus *Laccaria* play pivotal roles in forest ecosystems, specifically forming ectomycorrhizal relationships with the root systems of various plants. Some *Laccaria* species are recognized for their edibility, holding potential as a sustainable food source in the context of future food security and dietary diversification. In this study, the species diversity of *Laccaria* in Yunnan was investigated, and four novel species were identified. Their taxonomical positions and phylogenetic affinities were confirmed through phylogenetic analysis based on ITS, nrLSU, *tef*1-α, and *rpb*2 sequence data. Macro- and micro-morphological characteristics of the new species are also given here. *Laccaria brownii* sp. nov. has a dark to slightly desaturated orange pileus, stipe context broadly fistulose and soft orange, and relatively smaller cheilocystidia and pleurocystidia. *Laccaria orangei* sp. nov. has a hemispherical to paraboloid pileus, abundant narrowly clavate, flexuose, and branched cheilocystidia. *Laccaria ruber* sp. nov. pileus is red on the margin, clearly striate on the pileus surface, basidia clavate, mostly four-spored, rarely two-spored. *Laccaria stipalba* sp. nov. stipe surface is white, long sterigmata (4–13 μm × 2–3 μm), pleurocystidia narrowly clavate to subclavate, flexuose or mucronate, rarely branch. The descriptions, illustrations, and phylogenetic analysis results of the new taxa are provided. In addition, the new taxa are compared with closely related taxa.

## 1. Introduction

*Laccaria* belongs to the family *Hydnangiaceae*, order *Agaricales*, phylum *Basidiomycota*, and kingdom Fungi, and is known for its important role in forest ecosystems, where, as with all mycorrhizal fungi, species facilitate the exchange of nutrients between plants and soil [1,2,3]. Approximately 85% of the terrestrial plants form mycorrhizal associations with fungi [4], and species of *Laccaria* are an important member of them [5].

The genus is characterized by collybioid to omphaloid basidiomata; echinulate acyanophilous and inamyloid basidiospores; a convex, plane, or umbilicate, and hygrophanous pileus; and a clamp present in all parts of the basidiomata [6,7,8]. *Laccaria* species are found worldwide in association with both Angiosperms and Gymnosperms worldwide [2]; forming ectomycorrhizas with tree species, many of which are of major economic importance [9,10]. The genus includes over 100 species of ectomycorrhizal fungi, which form symbiotic relationships with more than 20 genera of plants [11,12,13].

Since the establishment of *Laccaria* by Berk. and Broome in 1883, the taxonomy of this genus has been a subject of significant interest and research among mycologists [14,15,16,17,18,19,20,21,22]. To date, species of *Laccaria* have been divided into two sub-genera (*L.* subgen. *Laccaria* Berk. and Broome, *L.* subgen. *Maritimae* (Bon) Pázmány); seven sections (*L*. sect. *Amethystinae* Bon, *L*. sect. *Bisporae* Pázmány, *L*. sect. *Laccaria* Berk. and Broome, *L*. sect. *Maritimae* Bon, *L.* sect. *Obscurae* Pázmány, *L.* sect. *Purpureobadia* Pázmány, *L*. sect. Violacei Pázmány); and three subsections (*L*. subsect. *Amethystinae* (Bon) Contu, *L*. subsect. *Bisporae* Contu and *L*. subsect. *Laccaria* Berk. and Broome) [23,24,25,26]. However, the data lack phylogenetic support, which has made the taxonomic study of *Laccaria* consistently perplexing.

The species diversity of *Laccaria* is important; firstly, the mutualistic relationships of *Laccaria* with plant roots help improve nutrient uptake and support plant growth [2]. Different *Laccaria* species have been found to have different preferences for host plants. Therefore, a diverse community of *Laccaria* species can contribute to the health and diversity of plant communities in a given ecosystem [2]. *Laccaria bicolor* (Maire) P.D. Orton and *L. japonica* Popa and K. Nara have been found to have potential for use in bioremediation, as they are capable of breaking down and detoxifying a range of organic pollutants in soil [27,28]. A diverse community of *Laccaria* species could, therefore, provide a more comprehensive suite of tools for the remediation of contaminated soils. Furthermore, *Laccaria* species have been found to produce a range of bioactive compounds with potential for use in medicine, biotechnology, and agriculture. Therefore, a diverse community of *Laccaria* species could provide a wider range of compounds for exploration and development. The diversity of *Laccaria* species is important for maintaining healthy ecosystems, promoting bioremediation, and supporting biotechnological research and development [29].

With the development of molecular biology, the number of *Laccaria* species described from Asia has been increasing [14,15,16]. So far, 41 species of *Laccaria* have been described in Asia [11,17,18,19,20,21,22,23,24,25], of which 56% (23/41) are native to China.

Yunnan’s biodiversity is reflected in its diverse ecosystems, ranging from tropical rainforests in the south to alpine meadows in the north. The province is home to many plant species, with studies indicating that it hosts one of the richest floras in the world, making Yunnan one of the richest regions in the world in terms of fungal resources [11,18,30]. In this study, four new species of *Laccaria* are described from Yunnan Province with morphological and molecular data. The discovery contributes to understanding the diversity of fungal species in the Lancang-Mekong River Basin.

## 2. Materials and Methods

### 2.1. Morphological Study

Specimens were collected from Puer City, Qujing City, and Zhaotong City, Yunnan Province, China. They were photographed in the field; important collection information was recorded [31], separately wrapped in aluminium foil, or kept in a plastic collection box and taken to the laboratory of the Fungal Diversity Conservation and Utilization Team in Northwest Yunnan (Dali University). The fresh basidiomes were macro-morphologically described on the same day of collection. Color identification was performed using the Color Hexa website (www.colorhexa.com) to assign codes. After thoroughly drying at 50 °C in a food drier [32], the specimens were stored in sealed plastic bags and deposited in the Herbarium of Cryptogams Kunming Institute of Botany, Academia Sinica (KUN-HKAS). Dried materials were sectioned under a stereo microscope, transferred onto slides, and mounted in a 5% KOH solution. The morphological structures were observed as described by reference [33,34,35]. For microscopic characters, anatomical and cytological characteristics such as basidia, basidiospores, and cystidia were observed and photographed using a Nikon ECLIPSE Ni-U microscope (Nikon, Tokyo, Japan) at magnifications of up to ×1000. For SEM studies, fragments of the lamellae of the dried material were taken, sputter coated with gold, and analyzed with a Hitachi S520 (Hitachi, Chiyoda, Japan). The notation [x/y/z] specifies that measurements were made on x basidiospores measured from y basidiomata of z collections. At least 50 basidiospores and 20 basidia were measured from one basidioma. Basidiospore dimensions are given as (a–) b–c (–d). Where “a” and “d” refer to the minimum and maximum values of all measurements, respectively, b–c presents the range of 95% of the measured values, and Q is the length/width ratio of basidiospores, Qm is the average Q of all basidiospores, and is given as Qm ± standard deviation.

### 2.2. DNA Extraction, PCR Amplification, and Sequencing

Macro-morphological studies were conducted following the protocols provided by Genomic DNA, which was extracted from dried specimens using the Ezup Column Fungi Genomic DNA extraction kit (Sangon, Shanghai, China) following the manufacturer’s protocol. Primer pairs for PCR were, respectively, ITS1/ITS4 [36], LR5/LR0R [37], *rpb*2-5F/*rpb*2-7cR [38], and EF1-983F/EF1-2218R [39]. ITS, LSU, *rpb*2, and *tef*1-α were amplified in 25 μL reactions containing 12.5 μL of 2× Taq Plus Master Mix II (Vazyme Biotech Co., Ltd., Nanjing, China), 9.5 μL of ddH_2_O, 1 μL and 10 μM of forward and reverse primers, and 1 μL of DNA. The PCR amplicons were sent to Sangon Biotech (Shanghai, China) for Sanger sequencing. Sequence reads were assembled in SeqMan II (DNA STAR Inc., Madison, WI, USA).

### 2.3. Sequence Alignment and Phylogenetic Analysis

The newly generated sequences were checked using the BioEdit Sequence Alignment Editor version 7.0.4 and assembled using SeqMan (DNAstar, Madison, WI, USA). The sequences were then blasted using the Basic Local Alignment Search Tool (https://blast.ncbi.nlm.nih.gov; 10 October 2024) against the GenBank database [40] to check the most closely related sequences. Reference sequences for 116 specimens representing 63 species were retrieved minimally adjusted by hand in BioEdit v.7.0.4 [41] first and then aligned using TrimAl (v. 1.2.59) [42].

Maximum likelihood (ML) analysis was performed separately for each locus, and the concatenated dataset using RAxML-HPC2 v. 8.2.12 [43] as implemented on the CIPRES portal [44], with the GTR + G model for both genes and 1000 rapid bootstrap (BS) replicates, the GTR + G model was obtained by MrModeltest 2.2. For Bayesian inference (BI), the best substitution model for each character set was determined with MrModeltest 2.2 [45] on CIPRES, using the Akaike information criterion. Bayesian analysis was performed using MrBayes ver. 3.2.7a [46], as implemented on CIPRES.

## 3. Results

### 3.1. Phylogenetic Analyses

A total of 56 new sequences (ITS, LSU, *rpb*2, and *tef*1-α) were generated for *Laccaria* species and deposited in GenBank (Table 1). The ITS-LSU-*rpb*2-*tef*1-α dataset includes 116 specimens related to 63 species. Phylogenetic analyses were conducted with a 5.8S + LSU, ITS1 + ITS2, *rpb*2 codon, *rpb*2 introns + *tef*1-α introns, and *tef*1-α codon concatenated matrix [37]. The concatenated matrix contained 3533 positions (1058 for 5.8S + LSU, 450 for ITS1 + ITS2, 1026 for *rpb*2 exons, 163 for *tef*1-α introns + *rpb*2 introns, and 836 for *tef*1-α exons). Based on previous phylogenies [2,10,11,17,18,19,20,21,22], species of the *Mythicomyces corneipes* (Fr.) Redhead and A.H. Sm. (AFTOLID972; ES11.10.2.A) were selected as the outgroup. In the ITS, LSU, *rpb*2, and *tef*1-α datasets, the models selected by mrModelTest were GTR + I + G for 5.8S + LSU and *tef*1-α codon, GTR + G for ITS1 + ITS2 and *rpb*2 codon, and GTR + G for *rpb*2 introns + *tef*1-α introns.

In MrBayes analyses, two runs of five chains each were run for 2,000,000 generations and sampled every 200 generations. Convergence was further evaluated by checking that the potential scale reduction factor (PSRF) statistic was close to 1 for all parameters. Moreover, the effective sample size (ESS) was much higher than 200 for all parameters. A clade was considered to be supported if showing a bootstrap support value (BS) ≥ 75% and/or a posterior probability (PP) ≥ 0.90. Trees were edited in FigTree version 1.4.0 and PowerPoint.

**Figure 1:**
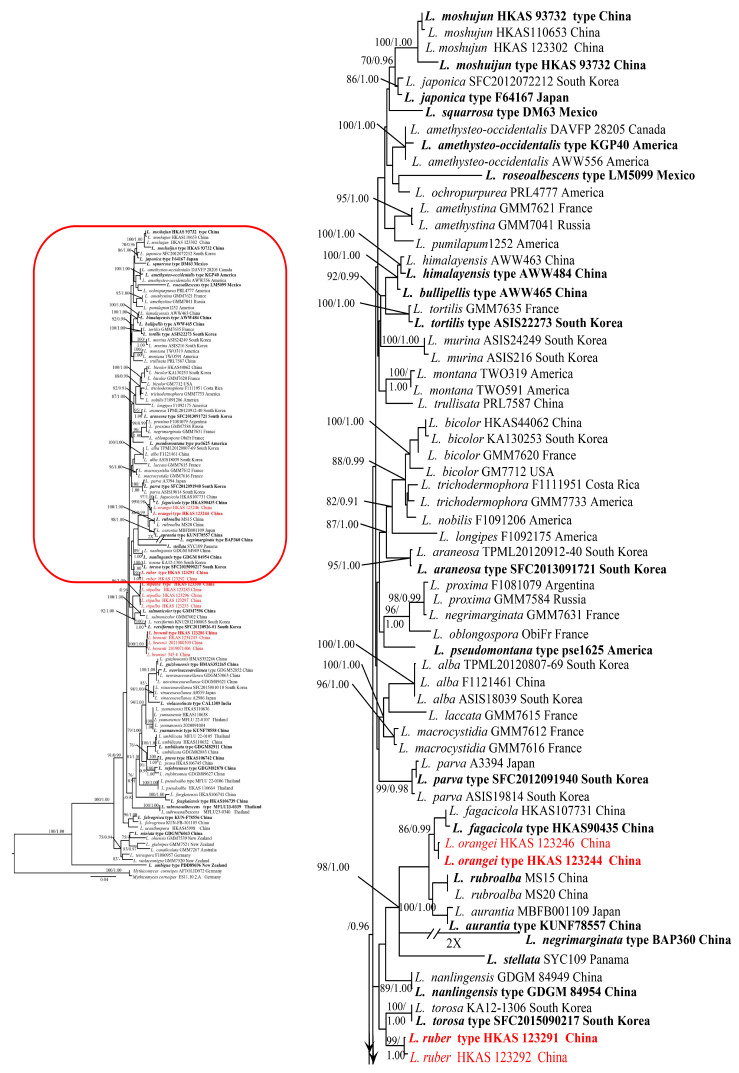

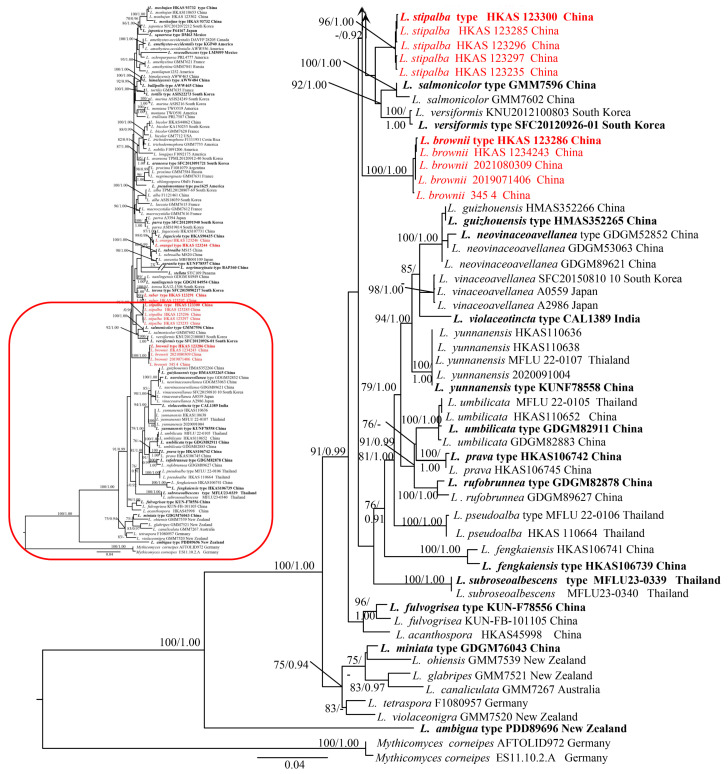
Maximum likelihood phylogeny of ITS1-5.8S-ITS2, LSU, *rpb*2, and *tef*1-α sequence data of *Laccaria*. ML bootstrap (≥70%) and posterior probabilities (≥0.90) are indicated above branches or in front of the branch leading to each node. The new species are highlighted in red; the holotype of each species is in bold.

The phylogeny from the combined datasets is presented in Figure 1. Fourteen specimens collected in southwestern China formed four monophyletic clades, described here as *L. brownii*, *L. orangei*, *L. ruber*, and *L. stipalba*, respectively. Each clade was well supported by both ML and BI analyses (Figure 1).

### 3.2. Taxonomy

***Laccaria brownii*** S.M. Tang, K.D. Hyde, and Z.L. Luo, sp. nov.

**Figure 2:**
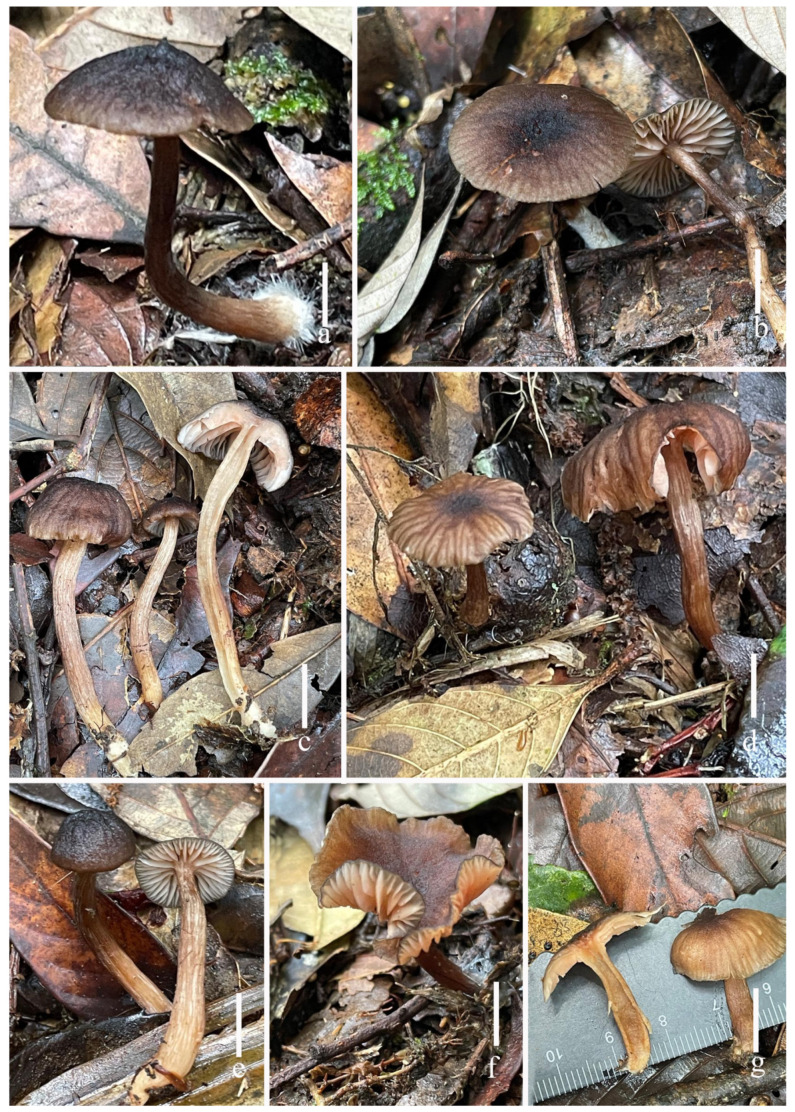
Fresh basidiomata of *Laccaria brownii* ((**a**,**b**) holotype, HKAS 123286, (**c**–**e**) HKAS 1234243, and (**f**,**g**) HKAS 144548). Scale bars = 1 cm.

**Figure 3:**
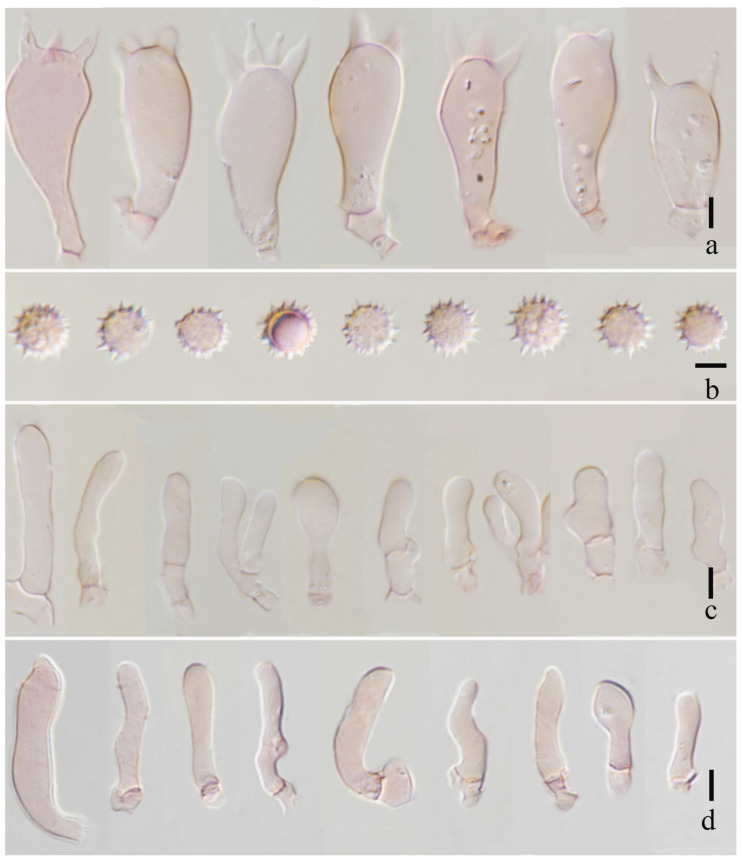
*Laccaria brownii* (holotype, HKAS 123286). (**a**) basidia; (**b**) basidiospores; (**c**) cheilocystidia; and (**d**) pleurocystidia. Scale bars = 10 μm.

**Figure 4:**
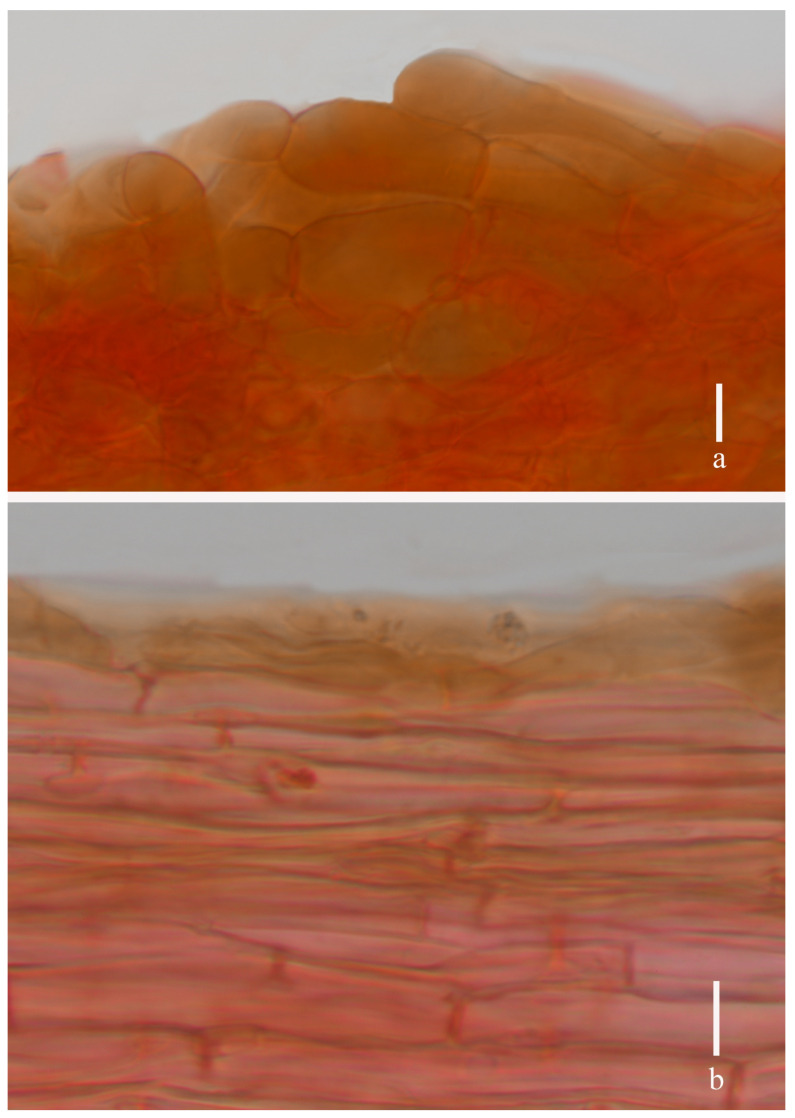
*Laccaria brownii* (holotype, HKAS 12328). (**a**) pileipellis; (**b**) stipitipellis. Scale bars = 10 μm.

**Figure 14:**
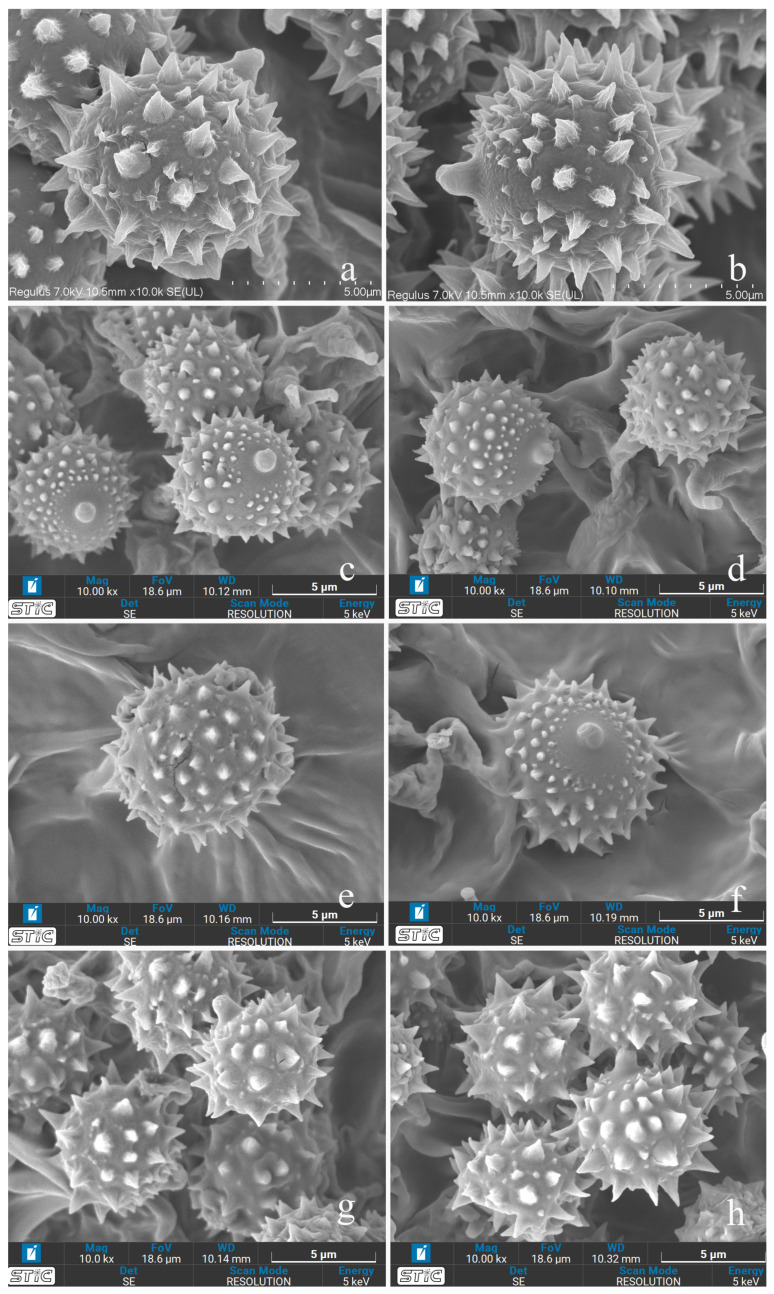
Characteristics of basidiospore ornamentations under scanning electron microscope (SEM). (**a**,**b**) *Laccaria brownii*; (**c**,**d**) *Laccaria orangei*; (**e**,**f**) *Laccaria ruber*; and (**g**,**h**) *Laccaria stipalba*.

Figure 2, Figure 3, Figure 4 and Figure 14a,b

**Fungal Name.** FN 572202

**Diagnosis**. *Laccaria brownii* is characterized by a slightly desaturated orange to brown pileus and stipe, soft orange lamellae, globose to subglobose basidiospores, pileipellis element by grayish inflate hyaline, narrowly clavate to subclavate cheilocystidia and pleurocystidia.

**Etymology**. The epithet “brownii” refers to the brown pileus and stipe of this fungus.

**Holotype**. China. Yunnan Province, Pu’er City, Ailao Mountains, 24°03′2.0″ N, 101°00′10″ E, elev. 2420 m, 3 August 2021, S.M. Tang, 2021080307 (HKAS 123286).

**Description**. ***Basidiomata*** medium size. ***Pileus*** 15–27 mm in diam., convex to hemispherical when young, plano-concave to concave, dark (#0a0a0a) at the center, slightly desaturated orange (#bd9b85) with a margin; a slightly depressed to depressed shape of center; margin inflexed when young, sometimes reflexed when old; context thin, 1–2 mm, soft orange (#f2d4bf), unchanging. ***Lamellae*** distant, segmentiform, subdecurrent, soft orange (#f2d4bf), 2–3 mm in height; lamella edge even or entire; lamellulae in 3–4 tiers. ***Stipe*** 20.8–39.2 × 3.0–4.3 mm, cylindrical, central, equal with an enlarged base and nearly subclavate, slightly desaturated orange (#bd9b85) to light grayish orange (#dac3b9), smooth, basal mycelium white (#ffffff); stipe context broadly fistulose, soft orange (#f2d4bf). ***Odor*** and taste not observed.

***Basidia*** 21–40 × 8–14 μm (mean length = 29.9 ± 5.6, mean width = 11.4 ± 1.8), clavate, mostly four-spored, rarely two-spored, sterigmata 4–8 μm × 1–3 μm (mean length = 6.0 ± 1.23, mean width = 2.8 ± 0.68). ***Basidiospores*** (excluding ornamentation) [105/3/2] 6.5–8.4× 6.1–7.7 μm (mean length = 7.3 ± 0.58, mean width = 7.1 ± 0.49), Q = 1.04–1.25, Qm = 1.04 ± 0.09, globose to subglobose, hyaline, echinulate, spines 1–2 μm long, ca. 0.6–1.1 μm wide at the base, crowded. ***Cheilocystidia*** 13–20 × 3–7 μm (mean length = 18 ± 4.8, mean width = 5.3 ± 1.4), narrowly clavate, thin-walled, colorless, and hyaline, abundant. ***Pleurocystidia*** 12–18 × 2–6 μm (mean length = 15 ± 2.2, mean width = 4 ± 1.1), narrowly clavate to subclavate, flexuose or mucronate, thin-walled, hyaline, abundant. ***Lamellar trama*** 80–120 μm thick, regular, composed of slightly thick-walled, filamentous hyphae 3–5 μm wide. Lamellar edge more in a number of sterile basidia. ***Subhymenium*** 8–11 μm thick, tightly interwoven, cellular, ramose, or irregular cells, 4–10 × 3–5 μm (mean length = 6 ± 2.1, mean width = 3.9 ± 0.6). Pileipellis 21–50 μm thick, grayish, inflated hyaline in KOH, composed of appressed, parallel, simply septate, thin-walled, cylindrical, filamentous hyphae 10–15 μm wide, colorless, and hyaline. ***Stipitipellis*** composed of appressed, parallel, simply septate, thick-walled, hyphae 3–8 μm wide; stipe trama composed of longitudinally arranged, grayish in KOH, clavate terminal cells, infrequently branching, septate, thick-walled hyphae hyaline, 3–10 μm wide. Caulocystidia not seen. ***Clamp connection*** present at some septa in pileipellis, lamellae, and stipitipellis.

**Habitat and phenology**. Scattered, gregarious, or caespitose on the ground in *Fagus*.

**Additional specimens examined**. China. Yunnan Province, Pu’er City, Ailao Mountains, N 24°03′2.0”, E 101°00′10”, elev. 2420 m, 3 August 2021, S.M. Tang, 2021080305 (HKAS 123243); *ibid.*, elev. 2322 m, 3 August 2021, S.M. Tang, 2021080309 (HKAS 144550); *ibid.*, elev. 2108 m, 6 August 2020, S.M. Tang, 2020080609 (HKAS 123243); *ibid.*, elev. 2112 m, 6 August 2020, S.M. Tang, 2020080610 (HKAS 144549); Zhaotong City, Yiliang County, Xiaocaoba Town, elev. 1825 m, 14 July 2019, S.M. Tang, 2019071406 (HKAS 144548).

**Notes**. In our multi-locus phylogeny, five specimens of *L. brownii* were clustered together with 100/1.00. *Laccaria murina* S. Imai and *L. anthracina* K. Wang, G.J. Li, Z. Du, and T. Z. Wei are similar to *L. brownii* in having gray to brown pileus. However, *L. murina* has relatively smaller basidiomata (pileus size 10–15 mm) and relatively larger basidiomata [47]. *Laccaria anthracina* has a relatively smaller basidiomata (pileus size 25–65 mm), and relatively larger basidiospores (7.0–9.5 × 7–9.0 μm) [48].

***Laccaria orangei*** S.M. Tang, K.D. Hyde, and Z.L. Luo, sp. nov.

**Figure 5:**
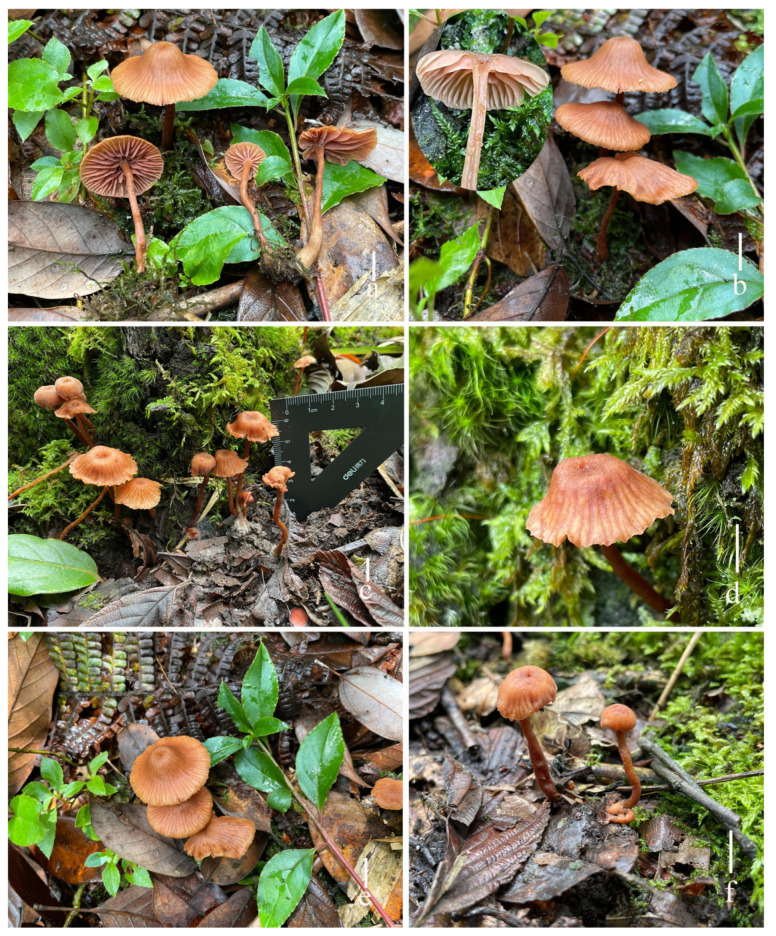
Fresh basidiomata of *Laccaria orangei* ((**a**,**b**) holotype, HKAS 123244), ((**c**–**f**) HKAS 123246). Scale bars = 1 cm.

**Figure 6:**
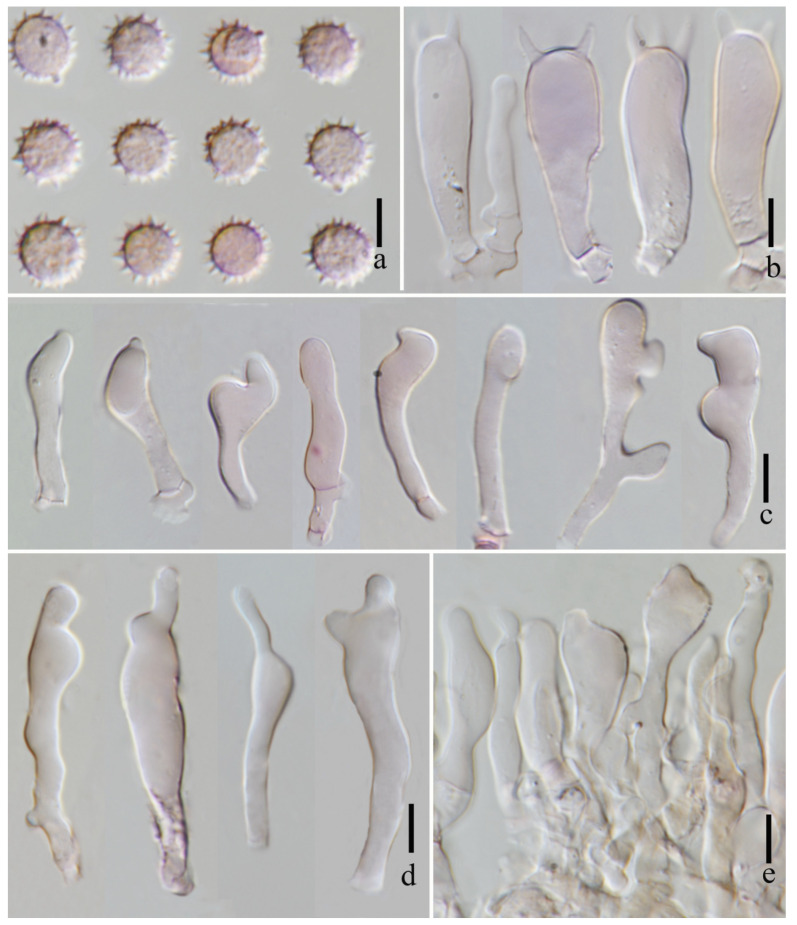
*Laccaria orangei* (holotype, HKAS 123244). (**a**) basidiospores; (**b**) basidia; (**c**) pleurocystidia; and (**d**,**e**) cheilocystidia. Scale bars = 10 μm.

**Figure 7:**
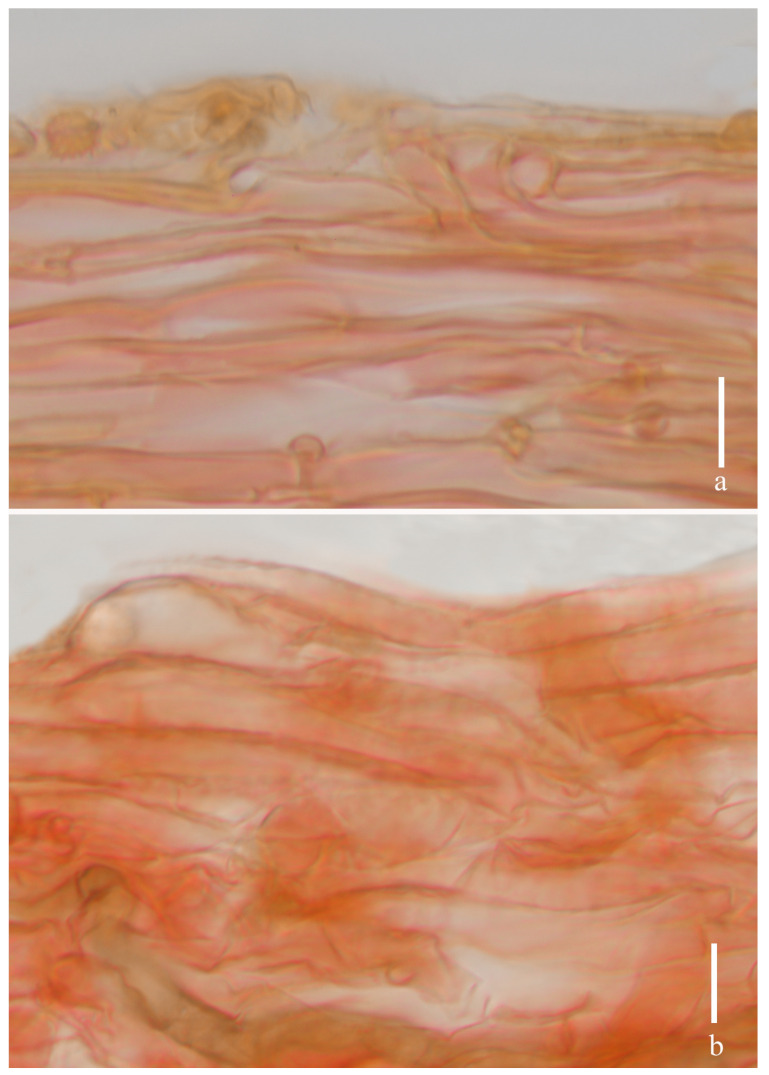
*Laccaria orangei* (holotype, HKAS 123244). (**a**) pileipellis; (**b**) stipitipellis. Scale bars = 10 μm.

Figure 5, Figure 6, Figure 7 and Figure 14c,d

**Fungal Names. FN 572019**

**Diagnosis.** *Laccaria orangei* is distinguished by its hemispherical to paraboloid pileus and soft orange basidiomata, narrowly clavate to subclavate, flexuose or mucronate of pleurocystidia, and narrowly clavate, flexuose branches of cheilocystidia.

**Etymology.** The epithet “orangei” refers to the orange pileus of this fungus.

**Holotype.** China, Yunnan Province, Zhaotong City, Yiliang County, Xiaocaoba Town, 27°75′ 81.3″ N, E 103°23′ 51.1″ E, elev. 1789 m, 24 July 2021, S.M. Tang, 2021072410 (HKAS 123244).

**Description. *Basidiomata*** medium size. ***Pileus*** 18–32 (–41) mm in diam., hemispherical to paraboloid, becoming campanulate with age, soft orange (#e7a582), unchanging, when dry moisture loss of moisture or with age becoming whitish, clearly striate on the surface; subumbonate of center; margin reflexed; context thin, 1–2 mm, slightly desaturated orange (#c59682), unchanging. Lamellae distant, arcuate, adnate with a decurrent tooth, soft orange (#f1c7b7), 3–5 mm in height; lamella edge even or entire; lamellulae in 2–3 tiers. ***Stipe*** 46.0–55.1 × 3.4–5.5 mm, cylindrical, central, equal with an enlarged base and nearly subclavate, soft orange (#e9ad88), smooth, basal mycelium white (1A1); stipe context fistulose, soft orange (#e9ad88). ***Odor*** and taste not observed.

***Basidia*** 30–40 × 7–10 μm (mean length = 35 ± 4.1, mean width = 8 ± 1.4), clavate, mostly two-spored, rarely four-spored, sterigmata 2–5 μm × 1–3 μm (mean length = 4.4 ± 1.1, mean width = 2.2 ± 0.82). ***Basidiospores*** (excluding ornamentation) [109/2/2] 5.8–8.0 × 5.5– 7.3 μm (mean length = 6.9 ± 0.58, mean width = 6.5 ± 0.49), Q = 1.00–1.29, Qm = 1.05 ± 0.11, globose to subglobose, hyaline, echinulate, spines 0.7–1.8 μm long, ca. 0.5–0.8 μm wide at the base, crowded. ***Cheilocystidia*** 20–49 × 4–5 μm (mean length = 34.5 ± 4.5, mean width = 4.8 ± 0.6), narrowly clavate, flexuose, branches, thin-walled, colorless, and hyaline, abundant. ***Pleurocystidia*** 15–30 × 5–7 (–10) μm (mean length = 33.6 ± 10.7, mean width = 6.5 ± 2.1), narrowly clavate to subclavate, flexuose or mucronate, thin-walled, hyaline, abundant. ***Lamellar trama*** 90–127 μm thick, regular, composed of slightly thick-walled, filamentous hyphae 2–8 μm wide. ***Lamellar edge*** more in many sterile basidia and pleurocystidia. ***Subhymenium*** 13–20 μm thick, tightly interwoven, cellular, ramose, or irregular cells, 5–9 × 3–6 μm (mean length = 7 ± 1.9, mean width = 4.4 ± 1.4). ***Pileipellis*** 30–60 μm thick, orange hyaline in KOH, composed of appressed, parallel, simply septate, thin-walled, cylindrical, filamentous hyphae 7–9 μm wide, colorless, and hyaline. Stipitipellis is composed of appressed, parallel, simply septate, thick-walled, hyphae 3–7 μm wide; stipe trama is composed of longitudinally arranged, pastel red in KOH, clavate terminal cells, infrequently branching, septate, thick-walled, hyphae hyaline 3–10 μm wide. ***Caulocystidia*** not seen. ***Clamp connection*** present at some septa in pileipellis, lamellae, and stipitipellis.

**Habitat and phenology**. Scattered, gregarious, or caespitose on the ground in the *Dipterocarpus* and *Fagus*.

**Additional specimens examined**. China, Yunnan Province, Zhaotong City, Yiliang County, Xiaocaoba Town, elev. 1689 m, 24 July 2021, S.M. Tang, 2021072410 (HKAS 123246); *ibid*., elev. 1521 m, 24 July 2021, S.M. Tang, 2021072405 (HKAS 123301); *ibid*., elev. 1782 m, 24 July 2021, C.C. Ao, A202124-1 (HKAS 123242); *ibid*., elev. 1643 m, 24 July 2021, L. Wang, W007 (HKAS 123248).

**Notes**. Phylogenetically, the species is closely related to *L. fagacicola* Yang-Yang Cui, Qing Cai, and Zhu L. Yang; *L. rubroalba* X. Luo, L. Ye, P.E. Mortimer, and K.D. Hyde; and *L. aurantia* Popa, Rexer, Donges, Zhu L. Yang, and G. Kost. *Laccaria fagacicola* differs from *L. orangei* by its convex to applanate, brownish orange to brownish pileus, relatively longer basidia (45–60 × 9–12 μm), and sterigmata (5–8 μm), pleurocystidia lacking and cheilocystidia filamentous to narrowly clavate [49], the ITS sequences differences between *L. orangei* (HKAS 123244, holotype) and *L. fagacicola* (HKAS 90435, holotype) were 0.78% (5/643, no gaps). *Laccaria rubroalba* has a reddish-white pileus, flexuous to narrowly cylindrical pleurocystidia, and cylindrical to capitate cheilocystidia [20]. *Laccaria aurantia* has relatively larger basidiomata (pileus size 35–40 mm in diam.), basidia (40–45 × 10–12 μm), and basidiospores (9–10 × 8–10 μm) [18].

*Laccaria acanthospora* A.W. Wilson and G.M. Muell. and *L. ambigua* K. Hosaka, A.W. Wilson, and G.M. Mueller are morphologically similar to *L. orangei* for having an orange pileus. *Laccaria acanthospora* is different by smaller basidiomata (pileus size 4–15 mm in diam.), and larger basidia (40–56 × 10–14 μm) [17]. *Laccaria ambigua*, originally reported from New Zealand North, has smaller basidiomata (pileus size 8–10 mm) and larger basidiospores (9–9.6 × 9.6–9.9 μm) [2].

***Laccaria ruber*** S.M. Tang, K.D. Hyde, and Z.L. Luo, sp. nov.

**Figure 8:**
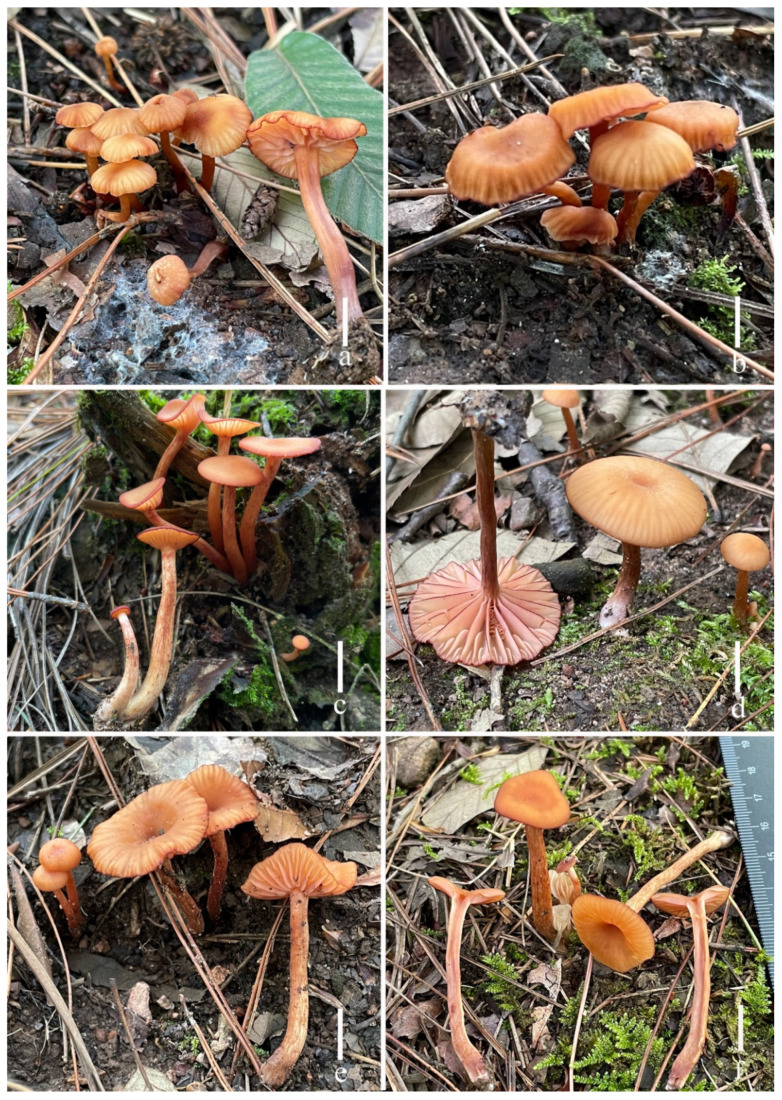
Fresh basidiomata of *Laccaria ruber* ((**a**,**b**) holotype, HKAS 123291, (**c**,**d**) HKAS 123292, (**e**,**f**) HKAS 123294). Scale bars = 1 cm.

**Figure 9:**
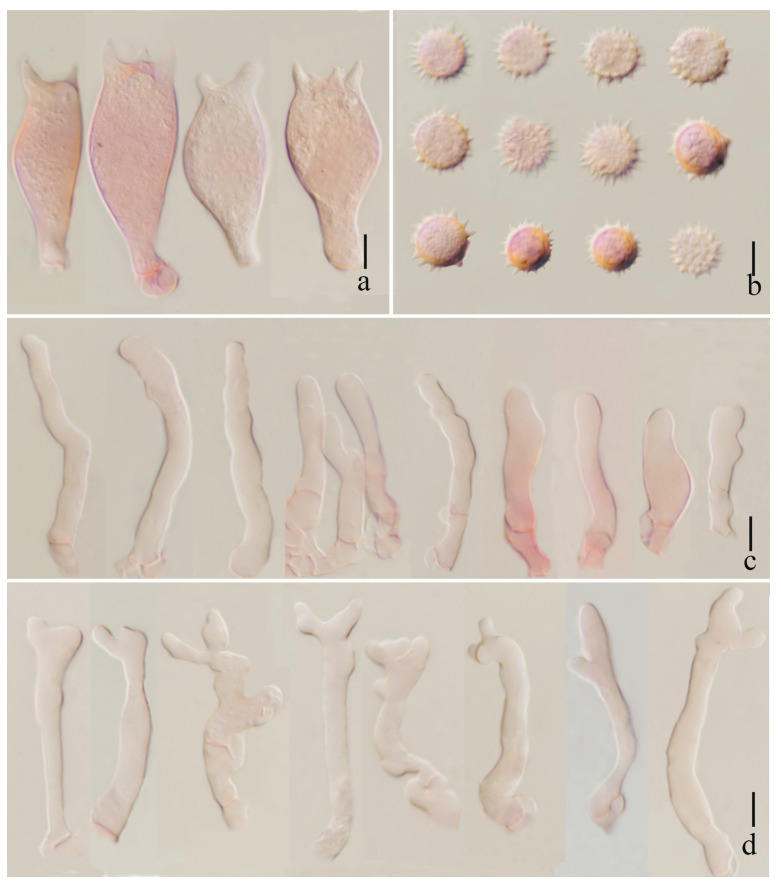
*Laccaria ruber* (holotype, HKAS 123291). (**a**) basidia; (**b**) basidiospores; (**c**) pleurocystidia; and (**d**) cheilocystidia. Scale bars = 10 μm.

**Figure 10:**
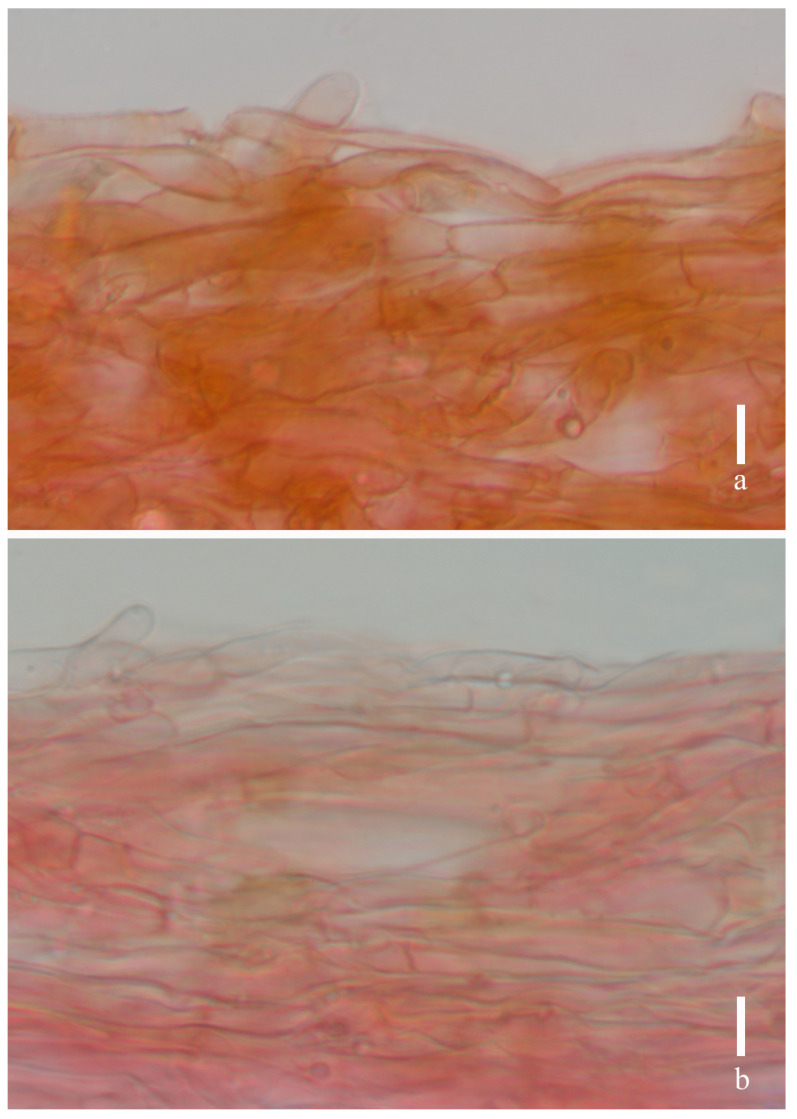
*Laccaria ruber* (holotype, HKAS 123291). (**a**) pileipellis; (**b**) stipitipellis. Scale bars = 10 μm.

Figure 8, Figure 9, Figure 10 and Figure 14e,f

**Fungal Names.** FN 572203

**Diagnosis.** *Laccaria ruber* is characterized by its soft orange basidiomata, which are narrowly clavate, flexuose, or mucronate, and branched cheilocystidia.

**Etymology.** The epithet “ruber” refers to the red margin of the pileus.

**Holotype**. China, Yunnan Province: Qujing City, Zhanyi County, elev. 2001 m, 30 July 2021, S.M. Tang (HKAS 123291).

**Description. *Basidiomata*** medium size. ***Pileus*** 18–24 mm in diam., convex to applanate, hemispherical, applanate to plano-concave, soft orange (#f2b790), when dry moisture loss of moisture or with age becoming whitish, clearly striate on the surface; depressed to subumbonate of center when young, becoming umbilicate with age; margin straight, clearly red (#c71e0c); and context thin, 1–2 mm, soft orange (#f2b790), unchanging. ***Lamellae*** distant, segmentiform to subventricose, adnate to narrowly adnate, soft orange (#ec9c7f), 1–3 mm in height; lamella edge even or entire, clearly red color; lamellulae in 3–4 tiers. ***Stipe*** 34.0–49.1 × 3.7–5.5 mm, cylindrical, central, equal with an enlarged base and nearly subclavate, soft orange (#f7a160), smooth, basal mycelium white (1A1); and stipe context broadly fistulose, soft orange (#f7a160). ***Odor*** and taste not observed.

***Basidia*** 33–39 × 10–14 (–17) μm (mean length = 35.6 ± 1.7, mean width = 13 ± 1.9), clavate, mostly four-spored, rarely two-spored, sterigmata 4–8 μm × 2–3 μm (mean length = 5.7 ± 1.9, mean width = 2.9 ± 0.47). ***Basidiospores*** (excluding ornamentation) [120/3/2] 7.2–10.3 × 7.3–9.4 (–10.8) μm (mean length = 8.6 ± 0.74, mean width = 8.2 ± 0.80), Q = 1.00–1.21, Qm = 1.04 ± 0.09, globose to subglobose, hyaline, echinulate, spines 1–2 μm long, ca. 0.8–1.2 μm wide at the base, crowded. ***Cheilocystidia*** 14–35 × 4–7 μm (mean length = 24.1 ± 4.1, mean width = 5.5 ± 1.1), narrowly clavate, flexuose or mucronate, branches, thin-walled, colorless, and hyaline, abundant. ***Pleurocystidia*** 18–48 × 3–5 μm (mean length = 35.8 ± 11, mean width = 4.5 ± 0.7), narrowly clavate to subclavate, flexuose or mucronate, thin-walled, hyaline, abundant. ***Lamellar trama*** 130–150 μm thick, regular, composed of slightly thick-walled, filamentous hyphae 4–7 μm wide. ***Lamellar edge*** more in several sterile basidia. ***Subhymenium*** 7–10 μm thick, tightly interwoven, cellular, ramose, or irregular cells, 4–7 × 3–6 μm (mean length = 6.1 ± 0.9, mean width = 4.6 ± 1.3). ***Pileipellis*** 100–130 μm thick, orange hyaline in KOH, composed of appressed, parallel, simply septate, thin-walled, cylindrical, filamentous hyphae 7–10 μm wide, colorless, and hyaline. ***Stipitipellis*** is composed of appressed, parallel, simply septate, thick-walled, hyphae 2–5 μm wide; stipe trama is composed of longitudinally arranged, pastel red in KOH, clavate terminal cells, infrequently branching, septate, thick-walled, hyphae hyaline 4–8 μm wide. ***Caulocystidia*** not seen. ***Clamp connection*** present at some septa in pileipellis, lamellae, and stipitipellis.

**Habitat and phenology.** Scattered or gregarious on the ground in *Fagus* and *Pinus* mixed forest.

**Additional specimens examined.** China. Yunnan Province, Qujing City, Zhanyi County, elev. 2005 m., 30 July 2021, S.M. Tang, 2021073021 (HKAS 123292, paratype); *ibid*., elev. 2018 m., 30 July 2021, C.C. Ao, A20210730-10 (HKAS 123293); *ibid*., elev. 2100 m., 29 July 2021, C.C. Ao, A20210729-05 (HKAS 123294); *ibid*., elev. 2100 m., 30 July 2021, S.M. Tang, 2021073008 (HKAS 123295).

**Notes**: In our phylogenetic analysis, *L. ruber* is sister to *L. torosa.* However, *L. torosa* differs by its orange-brown to brown pileus and lamellae, relatively larger basidia (39–47 × 12–17 μm), cheilocystidia filamentous to subclavate, no branch, and present abundant caulocystidia on the stipe surface. The ITS sequence differences between *L. torosa* (SFC 20150902-17, holotype) and *L. ruber* (HKAS 123291, holotype) were 2.95% (19/643, no gaps) [21].

*Laccaria acanthospora*, *L. ambigua*, *L. aurantia*, and *L. indohimalayana* K. Das, I. Bera, and Vizzini are similar to *L. ruber* in their orange basidiomata. However, *Laccaria acanthospora* has smaller basidiomata (pileus size 4–15 mm in diam.), relatively larger basidia (40–56 × 10–14 μm), and absent cheilocystidia and pleurocystidia [17]. *Laccaria ambigua* also has smaller basidiomata (pileus size 8–10 mm in diam.) and absent cheilocystidia and pleurocystidia [17]. *Laccaria aurantia* has larger basidiomata (35–40 mm) and basidia (40–50 × 10–12 μm), relatively longer stipe (80 mm) [18]. *Laccaria indohimalayana* has larger basidiomata (pileus size 40–95 mm, stipe 80–150 × 12–65 mm), smaller basidiospores (7.60–8.26 × 6.59–7.19 μm), and no cheilocystidia and pleurocystidia [50].

***Laccaria* *stipalba*** S.M. Tang, K.D. Hyde, and Z.L. Luo, sp. nov.

**Figure 11:**
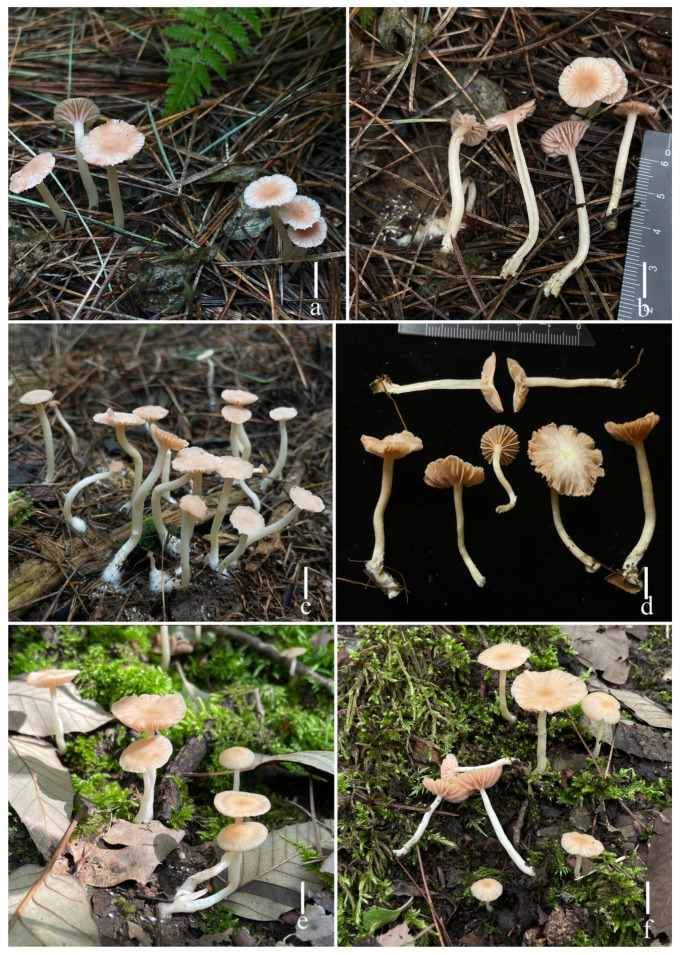
Fresh basidiomata of *Laccaria stipalba* ((**a**,**b**) holotype, HKAS 123300, (**c**,**d**) HKAS 123285, and (**e**,**f**) HKAS 123296). Scale bars = 1 cm.

**Figure 12:**
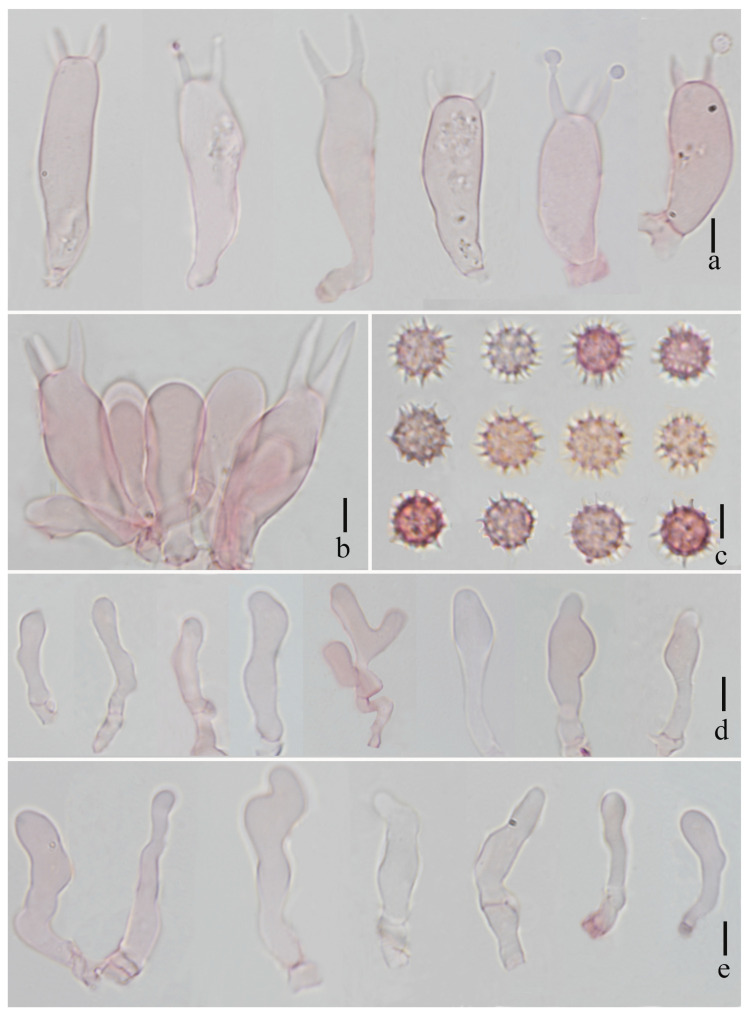
*Laccaria stipalba* (HKAS 123300, holotype). (**a**) basidia; (**b**) hymenium; (**c**) basidiospores; (**d**) pleurocystidia; and (**e**) cheilocystidia. Scale bars = 10 μm.

**Figure 13:**
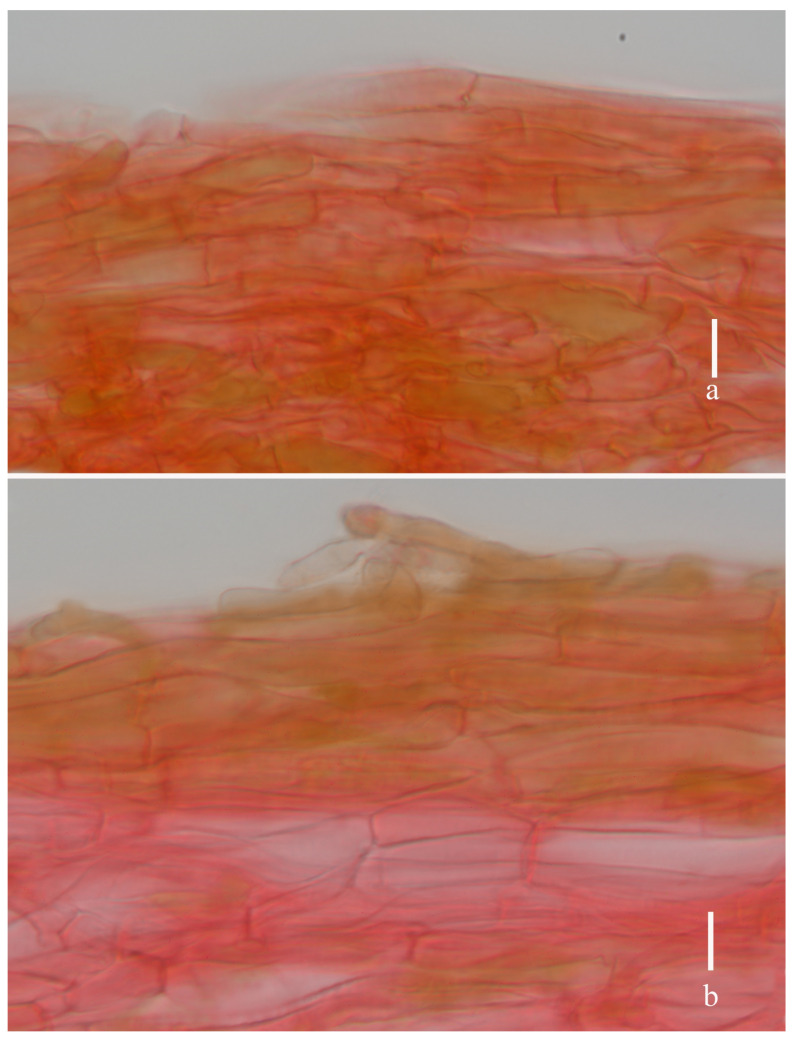
*Laccaria stipalba* (HKAS 123300, holotype). (**a**) pileipellis; (**b**) stipitipellis. Scale bars = 10 μm.

Figure 11, Figure 12, Figure 13 and Figure 14g,h

**Fungal Names.** FN 572204

**Diagnosis.** *Laccaria stipalba* is distinctive because of the white stipe, relatively longer sterigmata, and narrowly clavate, flexuose, or mucronate cheilocystidia.

**Etymology.** The epithet “stipalba” refers to the white stipe surface of the holotype.

**Holotype**. China, Yunnan Province. Zhaotong City, 27°27′ 52″ N, 103° 43′ 23″ E, elev. 2200 m., 23 July 2021, L. Wang, W2021-11 (HKAS 123300).

**Description.** Basidiomata medium size. Pileus 18–42 mm in diam., convex to applanate at young, becoming hemispherical, applanate to plano-concave with age, desaturated dark orange (#a6795f) at the center, light grayish pink (#e6e0e4) with margin, when dry moisture loss of moisture or with age becoming whitish, clearly striate on the surface; depressed of center; margin straight; context thin, 1–2 mm, pale orange light grayish pink (#e6e0e4), unchanging. Lamellae distant, broadly ventricose, adnexed, soft orange (#e7c89c), 2–3 mm in height; lamella edge even or entire; lamellulae in 1–2 tiers. Stipe 46.0–66.1 × 2.6–5.7 mm, cylindrical, central, white (#fcfcfc) to light grayish yellow (#e5e4ce), smooth, basal mycelium white (1A1); stipe context fistulose, white (#fcfcfc). Odor and taste not observed.

***Basidia*** 29–35 × 10–12 μm (mean length = 31.2 ± 1.6, mean width = 10.4 ± 1.2), clavate, mostly four-spored, rarely two-spored, sterigmata 6–13 μm × 2–3 μm (mean length = 9.5 ± 4.15, mean width = 3.0 ± 0.36). ***Basidiospores*** (excluding ornamentation) [150/3/2] 5.8–8.4 × 5.5–8.1 μm (mean length = 6.9 ± 0.68, mean width = 6.6 ± 0.67), Q = 1.00–1.29, Qm = 1.04 ± 0.13, globose to subglobose, hyaline, echinulate, spines 1–2 μm long, ca. 1–2 μm wide at the base, crowded. ***Cheilocystidia*** (10–) 20–30 × 4–7 μm (mean length = 24.0 ± 3.2, mean width = 5.1 ± 1.2), narrowly clavate, flexuose, or mucronate, thin-walled, colorless, and hyaline, abundant. ***Pleurocystidia*** 12–26 × 2–7 μm (mean length = 21 ± 5.1, mean width = 5 ± 1.5), narrowly clavate to subclavate, flexuose, or mucronate, rarely branch, thin-walled, hyaline, abundant. ***Lamellar trama*** 69–81 μm thick, regular, composed of slightly thick-walled, filamentous hyphae 2–8 μm wide. ***Lamellar edge*** more in number of sterile basidia and cheilocystidia. ***Subhymenium*** 7–10 μm thick, tightly interwoven, cellular, ramose, or irregular cells, 5–7 × 3–4 μm (mean length = 6 ± 1.2, mean width = 3.5 ± 0.5). ***Pileipellis*** (42–) 59–100 μm thick, colorless hyaline in KOH, composed of appressed, parallel, simply septate, thin-walled, cylindrical, filamentous hyphae 4–7 μm wide, colorless, and hyaline. ***Stipitipellis*** is composed of appressed, parallel, simply septate, thick-walled, hyphae 3–7 μm wide; stipe trama is composed of longitudinally arranged, colorless in KOH, clavate terminal cells, infrequently branching, septate, thick-walled, hyphae hyaline 3–10 μm wide. ***Caulocystidia*** not seen. ***Clamp connection*** present at some septa in pileipellis, lamellae, and stipitipellis.

**Habitat and phenology.** Scattered or gregarious on the ground in *Fagus* and *Pinus* mixed forest.

**Additional specimens examined.** China, Yunnan Province. Qujing City, Zhanyi County, elev. 2015 m, 29 July 2021, S.M. Tang, 2021072923 (HKAS 123296); *ibid*., elev. 2011 m, 30 July 2021, S.M. Tang, 2021073020 (HKAS 123297); *ibid*., elev. 2035 m, 30 July 2021, C.C. Ao, A2021073005 (HKAS 123235); Zhaotong City, elev. 2180 m, 23 July 2021, S.M. Tang, 2021072306 (HKAS 123285).

**Notes**: In our phylogenetic analysis, *L. stipalba* is close to *L. salmonicolor* and *L. versiformis*. However, *L. salmonicolor* has a reddish-brown to pale brown-buff pileus, shorter spines (1–3 μm), larger basidia (39–52 × 12–14 μm), and absent pileocystidia [17]. *Laccaria versiformis* has a pale brown to pale orange buff pileus and stipe, relatively larger basidiospores (7.5–10 × 7.5–9.5 μm), and basidia (41–55 × 10–14 μm) [21].

In the field, *L. stipalba* is easily confused with *L. alba* Zhu L. Yang and Lan Wang and *L. pseudoalba* S.M Tang and S.H. Li due to their similar orange-white to whitish basidiomata. However, *L. alba* has a relatively thicker pileipellis (30–75 μm), shorter sterigmata (1.5–2 μm), absent pleurocystidia, narrower cheilocystidia (4–6 μm), and clavate, hyaline caulocystidia [16]. *Laccaria pseudoalba*, originally reported from Thailand, has a stipe surface that is pale to pastel red; the ITS sequence difference between *L. pseudoalba* (MFLU 22-0106, holotype) and *L. stipalba* (HKAS 123300, holotype) is 6.38% (41/643, no gaps) [11].

## 4. Discussion

The taxonomic knowledge of *Laccaria* species in China, particularly in tropical and subtropical regions, is still nascent and requires further exploration. Historically, many Chinese specimens of *Laccaria* were misidentified as *L. amethystea* (Bull.) Murrill, *L. bicolor*, *L. laccata* (Scop.) Cooke and *L. vinaceoavellanea* based solely on morphological characteristics [11]. However, advancements in phylogenetic studies have enhanced our understanding of certain species within China, and the discovery of new species in *Laccaria* is rising [14,15,16,17,18,19,20,21,22]. Molecular data have been instrumental in the identification of species in this genus, which has led to a better appreciation of their diversity. Despite these advancements, the distribution and accurate identification of species such as *L. amethystea*, *L. bicolor*, and *L. laccata* in China necessitate further investigation. We will continue to collect specimens of the *Laccaria* from the Asian region and add species to the phylogeny of the *Laccaria*; the aim is to study the species diversity of *Laccaria* in Asia and to establish a phylogenetic framework for the genus, laying the groundwork for subsequent species research within *Laccaria*.

In our study, we found that several morphological features (see Table 2) of *Laccaria* species are important for species identification. These features include the pileipellis, stipitipellis, basidia, number, and length of sterigmata, basidiospore size, and surface ornamentation, as well as the size and shape characteristics of the cheilocystidia and pleurocystidia.

Some previous studies have described cheilocystidia and pleurocystidia in *Laccaria* species as non-existent; we speculate that some of these structures in *Laccaria* species may have been misidentified as terminal hyphae. This issue will be a focus of our future work, as the size and shape of the cheilocystidia and pleurocystidia are important for identified species of *Laccaria*. Most researchers have widely accepted the description of the narrowly clavate to subclavate structures on the hymenial surface as cystidia [16,17,18,19,20,21,30,49,51,52], rather than as terminal hyphae.

Recently, with the application of molecular systematics, the species of *Laccaria* in Asia have been updated [14,15,16,17,18,19,20,21,22]. Morphological examination and phylogenetic analyses identified 45 (see Table 2) species of *Laccaria* in Asia (including those introduced in this paper); these species are described from China (60%, 27/45), South Korea (26.7%, 12/45), Japan (15.6%, 7/45), India (15.6%, 7/45), and Thailand (3/45). While numerous *Laccaria* species have been documented in China, most of these reports originate from southwestern China.

**Figure 15:**
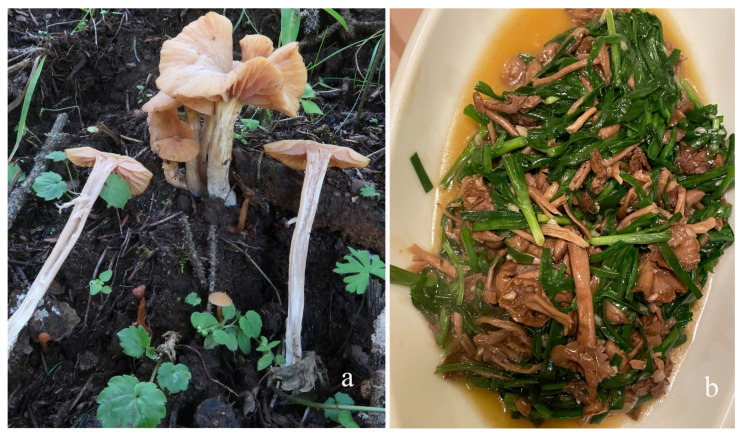
*Laccaria fengkaiensis* ((**a**) Growing in the wild; (**b**) as food after cooking).

Most basidiomata of larger *Laccaria* species, such as *L. yunnanensis*, *L. moshuijun*, and *L. fengkaiensis*, are highly valued for their culinary properties in Asia. They can be mixed with chili peppers, garlic, and chives for a stir-fry, creating an exceptional and delicious edible mushroom dish (Figure 15). These mushrooms are not only appreciated for their unique flavors but also their potential health benefits. In the subtropical broad-leaved forests of southern China, where these species are commonly found, they play a significant role in local gastronomy. Collecting these mushrooms is often a seasonal activity, reflecting the biodiversity and culinary traditions of the region [11].

The relationship between fungi, plants, and insects has always attracted the attention of researchers [55]. This fungus is often found in symbiotic relationships with plants, where it can enhance plant growth and resistance to pathogens. In a unique ecological interaction, the ectomycorrhizal fungus *Laccaria bicolor* has been shown to transfer nitrogen directly from soil-dwelling springtails (collembola) to white pine trees (*Pinus strobus*) [55,56,57]. This nitrogen transfer occurs specifically in white pine, as *L. bicolor* primarily associates with the roots of pine and spruce species in temperate forests. This process highlights the intricate symbiotic relationships between fungi, insects, and plants, where the fungus acts as a mediator to enhance nutrient acquisition for its host plant.

From an evolutionary perspective, *Laccaria* is known to form symbiotic relationships with a wide range of plant hosts. Initially, *Laccaria* species likely established symbiotic associations with angiosperms [2], which are characterized by their broad diversity and ecological dominance. Over time, these fungi expanded their host range to include both gymnosperms and angiosperms, reflecting an adaptive strategy to thrive in diverse environments. This evolutionary shift allowed *Laccaria* species to colonize a broader range of plant hosts, enhancing their ecological resilience and nutrient cycling capabilities [58]. For example, *Laccaria bicolor* has been shown to form ectomycorrhizal associations with various tree species, including *Pinus* (pine) and *Fagus* (beech), demonstrating the ability of these fungi to interact with both gymnosperms and angiosperms [59]. The ability to form symbiotic relationships with multiple plant species simultaneously further supports the idea that *Laccaria* have evolved to maximize their ecological fitness by adapting to different host environments. Further research is needed to confirm these associations and explore the specific mechanisms and benefits involved.

## Figures and Tables

**Table 1:** Names, sample numbers, and corresponding GenBank accession numbers of ITS, LSU, *rpb*2, and *tef*1-α sequences of the *Laccaria* taxa were newly generated in this study. “*” following a species name indicates that the specimen is the holotype. All specimen locations are in the Lancang-Mekong River Basin (Yunnan).

Species Name	Sample No.	GenBank Accession			
		ITS	LSU	*rpb*2	*tef*1-α
*Laccaria brownii*	HKAS 123286 *	PQ651574	PQ721003	PQ753337	PQ753351
*L. brownii*	HKAS 123243	PQ651575	PQ721004	PQ753338	PQ753352
*L. brownii*	HKAS 144548	PQ651576	PQ721005	PQ753339	PQ753353
*L. brownii*	HKAS 144549	PQ651577	PQ721006	PQ753340	PQ753354
*L. brownii*	HKAS 144550	PQ651578	PQ721007	PQ753341	PQ753355
*L. orangei*	HKAS 123244 *	PQ651572	PQ720997	PQ753335	PQ753349
*L. orangei*	HKAS 123246	PQ651573	PQ720998	PQ753336	PQ753350
*L. ruber*	HKAS 123291 *	PQ651570	PQ776317	PQ753333	PQ753347
*L. ruber*	HKAS 123292	PQ651571	PQ776318	PQ753334	PQ753348
*L. stipalba*	HKAS 123300 *	PQ651565	PQ753313	PQ753328	PQ753342
*L. stipalba*	HKAS 123285	PQ651566	PQ753314	PQ753329	PQ753343
*L. stipalba*	HKAS 123297	PQ651567	PQ753315	PQ753330	PQ753344
*L. stipalba*	HKAS 123296	PQ651568	PQ753316	PQ753331	PQ753345
*L. stipalba*	HKAS 123235	PQ651569	PQ753317	PQ753332	PQ753346

**Table 2:** The main morphological features and geographical distribution of known Asian *Laccaria* species. The newly added species in this study are in bold.

Species	Cap Size	Cap Color	Basidiospores Size	Cheilocystidia Size	Pleurocystidia Size	Distribution	References
*Laccaria acanthospora*	4–15 mm	Orange	7.0–10.0 × 7.0–10.0 μm	N/A	N/A	China	[17]
*L. angustilamella*	20–30 mm	Pinkish flesh-colored to buff	8.5–11.5 × 8.0–11.0 μm	11–66 × 3–7 μm	Absent	China	[16]
*L. alba*	10–35 mm	Pinkish to pale flesh	7.0–9.5 × 7.0–9.0 μm	20–48 × 4–6 μm	Absent	China; South Korea; Japan	[16,21]
*L. araneosa*	10–18 mm	Orange brown or light brown	8.0–9.0 × 7.4–9.0 μm	Absent	Absent	Japan; South Korea	[21]
*L. aurantia*	35–40 mm	Orange	9.0–10.0 × 8.0–10.0 μm	25–40 × 5–10 μm	25–40 × 5–10 μm	China; Japan	[18]
*L. bicolor*	15–60 mm	Pinkish flesh to reddish brown	6.5–8.0 × 6.0–7.5 μm	24–54 × 2–8 μm	N/A	China; South Korea	[17,21]
*L. bullipellis*	22 mm	Brown to orange brown	6.0–9.0 × 6.0–10.0 μm	N/A	50–62 × 6–29 μm	China	[17]
***L. brownii***	**15–27 mm**	**Dark at center, slightly desaturated orange**	**6.5–8.4× 6.1–7.7 μm**	**13–20 × 3–7 μm**	**12–18 × 2–6 μm**	**China**	**This study**
*L. cinnabarina*	10–90 mm	Dark brown to orange red	7.0–9.5 × 7.0–9.0 μm	Absent	Absent	China	[51]
*L. fagacicola*	20–45 mm	Orange brown or brown	7.0–9.0 × 6.5–8.0 μm	15–50 × 3–8 μm	Absent	China	[49]
*L. fengkaiensis*	50–90 mm	Orange white, pale white when young; light orange, pale red to pastel red with age	5.2–6.3 × 5.1–6.3 μm	Absent	Absent	China	[22]
*L. fulvogrisea*	<30 mm	Grey to reddish brown	8.0–10.0 × 8.0–11.0 μm	30–50 × 3–7 μm	30–50 × 3–7 μm	China	[18]
*L. griseolilacina*	20–35 mm	Light grayish to orange brown	8.0–10.8 × 8.2–10.9 μm	Absent	20–31 × 4–8 μm	South Korea	[52]
*L. guizhouensis*	15–63 mm	Flesh-colored to brownish white to brown	7–8 × 6–8 μm	20–35 × 3–6 μm	27–46 × 3–7 μm	China	[53]
*L. himalayensis*	6–34 mm	Brown to orange pink	6.5–10.0 × 6.5–10.0 μm	N/A	N/A	China	[17]
*L. indohimalayana*	40–95 mm	Brownish orange to light orange	6.9–8.3 × 6.6–8.1 μm	19–29 × 4–6 μm	15–39 × 3–5 μm	India	[50]
*L. japonica*	10–30 mm	Bright violet to purple, pale tan to flesh color	9.0–10.0 × 7.0–9.0 μm	30–50 × 3–7 μm	30–50 × 3–7 μm	Japan; South Korea; China	[21]
*L. longistriata*	5–45 mm	Brownish to brown at the center, cream, flesh-colored to brownish toward the margin	6.5–8.0 × 6.0–8.0 μm	Absent	30–60 × 4–8 μm	China	[51]
*L. laccata*	10–52 mm	Grayish purple to buff	7.5–9.5 × 7.5–9.5 μm	34–86 × 2–12 μm	Absent	India	[54]
*L. macrobasidia*	15–45 mm	Orange brown to light brown	8.7–11.4 × 7.9–10.3 μm	Absent	19–27 × 4–7 μm	South Korea	[52]
*L. moshuijun*	30 mm	Violet to bluish	8.0–9.0 × 9.0–10.0 μm	30–50 × 5–8 μm	30–50 × 3–6 μm	China	[44]
*L. montana*	7–35 mm	Reddish orange to brown	8.5–13.0 × 8.0–10.5 μm	N/A	N/A	India	[45]
*L. murina*	45 mm	Dark reddish brown to reddish orange brown	13.5–17.0 × 7.5–9.5 μm	N/A	N/A	Japan; South Korea; India	[21,45]
*L. nanlingensis*	30–55 mm	Brownish orange to brownish red	6.5–7.5 × 6–7 µm	40–60 × 4–6 µm	Absent	China	[10]
*L. negrimarginata*	5–15 mm	Orange brown to buff, blackish brown to dark brown	7.0–10.0 × 6.0–10.0 μm	N/A	N/A	China	[17]
*L. neovinaceoavellanea*	15–40 mm	Purplish pink to pale violet	7.0–8.0 × 7.0–8.0 μm	25–50 × 4–8 μm	Absent	China	[10]
*L. nobilis*	20–50 mm	Pale pinkish orange to dark orange	5.4–9.5 × 5.0–7.8 μm	29–44 × 1–4 μm	N/A	China	[46]
*L. olivaceogrisea*	9–32 mm	Grey	7.5–9.0 × 8.0–9.0 μm	Absent	Absent	India	[18]
***L. orangei***	**18–32 mm**	**Soft orange**	**5.8–8.0 × 5.5–7.3 μm**	**20–49 × 4–5 μm**	**15–30 × 5–7 μm**	**China**	**This study**
*L. pallidorosea*	10–25 mm	Brown to pink at center, becoming cream to white	7.0–9.0 × 6.5–8.5 μm	25–40 × 3–8 μm	Absent	China	[49]
*L. parva*	5–25 mm	Bright brown	8.0–10.0 × 8.5–10.0 μm	19–40 × 4–6 μm	25–33 × 4–6 μm	Japan; South Korea; China	[21]
*L. prava*	35–75 mm	Pastel red, pale red to reddish white	6.5–7.5 × 7.0–8.0 μm	Absent	Absent	China	[22]
*L. pseudoalba*	9–15 mm	Pale orange to orange white	6.9–11.3 × 6–10.4 μm	20–31 × 6–9 μm	15–31 × 6–8 μm	Thailand	[11]
*L. pumila*	10–35 mm	Orange brown to fading lighter	11.5–13.0 × 10–11.5 μm	N/A	N/A	India	[43]
*L. rubroalba*	22–40 mm	Reddish white	6.0–9.0 × 5.0–7.0 μm	12–26 × 5–9 μm	25–40 × 4–6 μm	China	[20]
***L. ruber***	**18–24 mm**	**Soft orange**	**7.2–10.3 × 7.3–9.4 μm**	**14–35 × 4–7 μm**	**18–48 × 3–5 μm**	**China**	**This study**
*L. rufobrunnea*	12–35 mm	Brownish orange to brownish red	8–9 × 7–8 µm	35–50 × 3–7 μm	Absent	China	[10]
*L. salmonicolor*	10–35 mm	Reddish brown	7.5–10.0 × 7.5–10.0 μm	N/A	N/A	China	[17]
*L. spinulosa*	8–25 mm	Brownish orange to brown	9–11.0 × 9–10.5 μm	Absent	Absent	China	[51]
***L. stipalba***	**18–42 mm**	**Light grayish pink**	**5.8–8.4 × 5.5–8.1 μm**	**20–30 × 4–7 μm**	**12–26 × 2–7 μm**	**China**	**This study**
*L. subroseoalbescens*	2–8 mm	Pale yellow to light yellow	7.0–8.9 × 6.8–9.0 μm	23–37 × 4–8 μm	36–59 × 5–8 μm	Thailand	[11]
*L. torosa*	10–70 mm	Orange brown or brown	8.0–9.0 × 8.0–9.5 μm	54–94 × 5–9 μm	55–75 × 7–13 μm	South Korea	[21]
*L. tortilis*	<30 mm	Orange brown to pinkish flesh	N/A	N/A	N/A	South Korea	[21]
*L. umbilicata*	10–28 mm	Pale yellow, pale orange to light orange	8–10 × 8–10 µm	30–47 × 3–8 µm	Absent	China	[10]
*L. versiformis*	10–35 mm	Pale brown to brown	7.5–8.2 × 7.5–9.5 μm	42–54 × 6–8 μm	42–65 × 7–9 μm	South Korea	[21]
*L. vinaceoavellanea*	N/A	Brownish vinaceous	7.4–9.2 × 7.4–9.2 μm	Absent	N/A	Japan; South Korea	[11,21]
*L. violaceotincta*	4–38 mm	Dark brown at the center, reddish brown towards the margin when young	7.0–8.0 × 7.0–8.0 μm	19–104 × 4–10 μm	Absent	India	[8]
*L. yunnanensis*	60–100 mm	Brown to flesh-colored	8.0–9.0 × 8.0–10.0 μm	30–50 × 4–6 μm	55–65 × 15–25 μm	China; Thailand	[11,18]

Annotation: “N/A” refers to not mentioned in the original description.

## Data Availability

The data presented in this study are openly available in Species Diversity of Edible MushroomsⅠ–Four New Laccaria Species from Yunnan Province, China at 10.6084/m9.figshare.28022549.
